# RNA-Seq Analyses Generate Comprehensive Transcriptomic Landscape and Reveal Complex Transcript Patterns in Hepatocellular Carcinoma

**DOI:** 10.1371/journal.pone.0026168

**Published:** 2011-10-17

**Authors:** Qichao Huang, Biaoyang Lin, Hanqiang Liu, Xi Ma, Fan Mo, Wei Yu, Lisha Li, Hongwei Li, Tian Tian, Dong Wu, Feng Shen, Jinliang Xing, Zhi-Nan Chen

**Affiliations:** 1 State Key Laboratory of Cancer Biology, Cell Engineering Research Center and Department of Cell Biology, Fourth Military Medical University, Xi'an, China; 2 Systems Biology Division, Zhejiang–California International Nanosystems Institute (ZCNI), Zhejiang University, Hangzhou, China; 3 Department of Urology, University of Washington, Seattle, Washington, United States of America; 4 Institute of Life Science and Biotechnology, Beijing Jiaotong University, Beijing, People's Republic of China; 5 Department of Comprehensive Treatment, Eastern Hepatobiliary Surgery Hospital, Second Military Medical University, Shanghai, People's Republic of China; Tsan Yuk Hospital, Hospital Authority, China

## Abstract

RNA-seq is a powerful tool for comprehensive characterization of whole transcriptome at both gene and exon levels and with a unique ability of identifying novel splicing variants. To date, RNA-seq analysis of HBV-related hepatocellular carcinoma (HCC) has not been reported. In this study, we performed transcriptome analyses for 10 matched pairs of cancer and non-cancerous tissues from HCC patients on Solexa/Illumina GAII platform. On average, about 21.6 million sequencing reads and 10.6 million aligned reads were obtained for samples sequenced on each lane, which was able to identify >50% of all the annotated genes for each sample. Furthermore, we identified 1,378 significantly differently expressed genes (DEGs) and 24, 338 differentially expressed exons (DEEs). Comprehensive function analyses indicated that cell growth-related, metabolism-related and immune-related pathways were most significantly enriched by DEGs, pointing to a complex mechanism for HCC carcinogenesis. Positional gene enrichment analysis showed that DEGs were most significantly enriched at chromosome 8q21.3–24.3. The most interesting findings were from the analysis at exon levels where we characterized three major patterns of expression changes between gene and exon levels, implying a much complex landscape of transcript-specific differential expressions in HCC. Finally, we identified a novel highly up-regulated exon-exon junction in *ATAD2* gene in HCC tissues. Overall, to our best knowledge, our study represents the most comprehensive characterization of HBV-related HCC transcriptome including exon level expression changes and novel splicing variants, which illustrated the power of RNA-seq and provided important clues for understanding the molecular mechanisms of HCC pathogenesis at system-wide levels.

## Introduction

Hepatocellular carcinoma (HCC) is one of the most common malignancies worldwide with an annual incidence of about 600,000 cases, 55% of which are in China [1]. The 5-year overall survival rate of individuals with HCC is relatively low, which has barely been improved over the past two decades. A better understanding of the molecular pathways that are active in HCC using a more comprehensive approach would potentially provide new strategies for clinical prevention and therapy.

Currently, several global approaches including array-based comparative genomic hybridization (CGH) [2], expression profiling based on DNA microarrays [3], proteomics based on 2D electrophoresis (2DG) and/or mass spectrometry (MS) [4] have been used to detect the changes at different molecular levels (DNA, RNA, or protein) in HCC, such as chromosomal imbalance and genetic instability, epigenetic alteration, gene expression, and gene regulation and translation. In particular, microarray-based gene profiling is the most commonly used method for studies on HCC including comparative analysis of cancer *vs.* non-cancerous samples [3], early stage *vs.* late stage [5], good prognosis *vs.* poor prognosis [6], and HCC patients with HBV *vs.* those with HCV infection [7]. However, array-based expression profiling has several limitations including incapability of detecting gene fusions and novel alternative splicing. Array analysis is further complicated by inconsistencies largely caused by differences in the platforms and compromised by its limited sensitivity in detecting lowly expressed genes [8], [9].

With the advance of the next-generation sequencing technologies, RNA-seq has become a useful tool in defining the transcriptomes of cells with the advantage of analyzing expression at exon levels as well as delineating novel splicing variants [10], [11]. Early application of RNA-seq included expression profiling of yeast [12], mouse brain, liver and skeletal muscle tissues [13], human embryonic kidney and a B cell line [14]. RNA-seq has several advantages over other expression profiling technologies including higher sensitivity, ability to detect splicing isoforms and somatic mutations [11]. For cancer expression profiling, Berger et al. [15] applied RNA-seq to expression profiling of melanoma. They identified 11 novel melanoma gene fusions and 12 novel read-through transcripts, providing an example of novel avenues for target discovery in cancers. To date, the RNA-seq analysis of HBV-related HCC has not been published. We therefore applied RNA-seq technology to analyze 10 matched pairs of HCC tissues and their adjacent non-cancerous tissues.

## Methods

### Ethics

This study was approved by the institutional review boards of the Forth Military Medical University. Written informed consent with a signature was obtained from each patient.

### Patients and Tissue Specimens

A total of 80 paired fresh-frozen tissue samples (cancer and matched adjacent non-cancerous tissue) were collected from Chinese HBV-related HCC patients undergoing surgery during the period of August 2009 to March 2010 in the Eastern Hepatobiliary Surgery Hospital of Shanghai, China. Diagnosis of all HCC cases was histologically confirmed by two independent pathologists and all tumor tissues was assessed by HE staining and only those with the percentage of tumor cells more than 90% and without necrosis were used for the analysis. Among HCC cases, 10 paired samples were randomly selected for RNA-sequencing. All these 10 patients were HBV positive. The clinical features of the patients were listed in Table S1.

### RNA preparation and sequencing

Total RNAs were extracted, according to the manufacturer's instruction, from about 60 mg of tissues for each of the 10 paired samples using the mirVana™ miRNA Isolation Kit (Ambion Inc.), which is also suitable for total RNA extraction. The RNA yields were quantified by NanoDrop ND1000 (Thermo- Fisher Scientific, Waltham, MA) and the RNA quality was assessed by the Agilent 2100 Bioanalyzer (Agilent, Santa Clara, CA). The RNA integrity number (RIN) of every RNA sample used for sequencing was more than 8. The cDNA libraries for 10 paired samples were constructed using mRNA-Seq Sample Prep Kit based on the Illumina Inc.'s guide. In brief, polyA-containing mRNA was purified using oligo-dT beads from 10 ug of total RNAs for each sample and fragmented into small fragments using divalent cations under elevated temperature. The cleaved RNA fragments were reverse-transcribed into first strand cDNA using random primers (Invitrogen Inc.), followed by second-strand cDNA synthesis. After end-repair processing, a single ‘A’ base was added to cDNA fragments at 3′ end. The cDNAs were then ligated to adapters, purified by 2% agrose gel, and then enriched by PCR to create the final cDNA library. Finally, RNA single-end sequencing was performed using Solexa/Illumina Genome Analyzer II using the standard protocol. The cDNA library of each sample was loaded to a single lane of an Illumina flow cell. The image deconvolution and calculation of quality value were performed using Goat module (Firecrest v.1.4.0 and Bustard v.1.4.0 programs) of Illumina pipeline v.1.4. For the 10 paired samples, 8 pairs were sequenced using one lane, one pair using two lanes and another pair using three lanes to assess the sequencing depth. Sequenced reads were generated by base calling using the Illumina standard pipeline. Each lane produced an average 20 million of 36-mer raw sequence reads.

### Alignment of sequenced reads

All alignments were performed using a tool package SOAP2 [16], which was developed professionally for short oligonucleotide analysis, allowing up to 2 mismatches with the references. Sequenced reads were aligned to human transcript reference sequences from the ENSEMBL database (Homo_sapiens.GRCh37.55.cdna.all.fa) for the expression analysis at gene/transcript levels, and were aligned to genome sequence and known exon-exon junction database from ENSEMBL for the expression analysis at exon level. Reads that were unable to be mapped to transcriptome were also aligned to novel exon-exon junction database that was previously developed by our group for detection of new alternative splicing events [17].

### Evaluation of data quality and sequencing depth

To test the reproducibility of sequencing, the correlation of gene expression between replicates of one sample was determined by Pearson correlation coefficient (PCC). Similar analyses were also performed to investigate the disparity of cancer and adjacent non-cancerous tissues. To assess the effect of sequencing depth for transcriptome analysis, we also determined the ability of increased sequencing raw reads for the identification of additional genes using a random sampling approach. Firstly, all raw reads of each repeat sequencing runs were pooled together for samples A39C and A39P. Then, 13 different bins of raw reads ranging from 5 to 65 million (using a step increase of 5 million) were randomly selected from pooled A39C or A39P, which were sequenced using three lanes in a flow cell. Each bin of random selected reads was mapped to transcriptome and the number of matched genes was tabulated for each bin and plotted.

### Differential expression analysis of gene, exon and novel exon-exon junction

After alignment to the transcriptome, the expression level of genes was determined based on the value of RPKM (reads per kilobase per million), which was calculated as the number of reads mapped to the transcripts of one gene divided by the transcript length and the number of total mapped reads in one sample [13]. For reads with multiple alignments, we arbitrarily assigned them to the transcript with the highest expression levels (read counts) under the assumption that the probability that the read is coming from the more abundantly expressed gene is higher than from the less abundant genes. For exon level expression analysis, reads were aligned onto genome firstly, and then unaligned reads were aligned onto the known exon-exon junction database from ENSEMBL. The expression level of each exon or exon-exon junction was also evaluated by RPKM, similarly calculated as the number of reads mapped to unique exon divided by the exon length and the number of total mapped reads in one sample. For reads with multiple alignments, we assigned the reads to every mapped exons if the read has ≤30 genome hits, while we discarded them if the alignment hits more than 30 exons. The number 30 was chosen to allow reads from gene families, of which are quite big (e.g. olfactory receptor family), to be counted while removing those highly abundant repeat sequence reads. For the reads mapped to exon-exon junction, we added counts to each junctional exon.

We performed principle component analysis of the RNA-seq data using RPKM values of all genes for all samples. The first principle component accounts for more than 86.5% of the variation. Furthermore, a heat map generated from unsupervised clustering analysis for the expression of all genes revealed that our HCC samples had no obvious heterogeneous classes or major subtypes (data not shown). Therefore, paired difference tests including the paired *t*-test and the paired wilcoxon signed rank test were applied to estimate the significance of expression difference based on RPKM value. In addition, edgeR [18], a recently developed software package specifically designed for analyzing RNA-seq data, was also used. A difference with *P*<0.05 for paired *t*-test and paired wilcoxon signed rank test and FDR (false discovery rate) <0.05 for edgeR was considered as significant. To assess the distribution of differentially expressed genes (DEGs) at different expression abundance levels, MA-plot was made based on the log_2_-transformed fold change (FC) of expression between HCC and adjacent non-cancerous tissues and log_2_-transformed average expression level for each gene across all samples.

### Comparison between RNA-seq and public microarray data

A public microarray dataset (GSE22058) was retrieved from the GEO database. The reason why we chose this dataset is that it is the most recent and the only large-scale dataset for the comparison of 96 HBV-related HCC and 96 adjacent non-cancerous liver tissues from Chinese patients [19], which are comparable to our data derived from HBV-infected Chinese populations. The log_2_-transformed average gene expression levels for common genes (mapped by common refseq IDs) were used to calculate the PCC values between the HCC tissues and the adjacent non-cancerous tissues. The scatter diagram was also plotted to assess the dynamic range of the gene expression level for both platforms.

### Functional annotation and positional gene enrichment analysis

The top 30 DEGs were analyzed with the gene annotation database at NCBI and the PubMed literature for their relationship with tumorigenesis. Additionally, three public databases of HCC, OncoDB.HCC (http://oncodb.hcc.ibms.sinica.edu.tw), HCCnet (http://www.megabionet.org/hcc/index.php), and EHCO (Encyclopedia of Hepatocellular Carcinoma genes Online, http://ehco.iis.sinica.edu.tw) were used to assess the differential expression of the DEGs in HCC-related datasets. Positional Gene Enrichment [20] (PGE, http://homes.esat.kuleuven.be/~bioiuser/pge/), a tool for the identification of over-represented chromosomal regions in a given gene set., was used to survey the chromosomal regions that are significantly enriched by differentially expressed genes (DEGs). Statistical significance was evaluated by an FDR value of <0.001. The Ingenuity Pathways Analysis (IPA) software package (Ingenuity Systems, www.ingenuity.com) was used to identify enriched bio-function terms and canonical pathways using gene symbols. Fisher's exact test of *P*<0.05 was considered significant. GSEA (Gene Set Enrichment Analysis) [21] was also used to identify enriched gene sets. To investigate the relationship of differential expressions at gene and exon levels, we plotted the average read counts for each exon of 5,288 DEE-containing genes in HCC tissues and adjacent non-cancerous tissues. Based on the plots of read coverage, the exon expression pattern was empirically classified.

### Identification of novel differentially expressed exon-exon junctions

We explored the possibility of RNA-seq in identifying novel spliced transcripts consisting of novel splicing events of known annotated exons. Several programs were available for such types of analysis including QPALMA [22], Splicemap [23], Mapsplice [24], TopHat [25], GSNAP [26] and PALMapper [27]. QPALMA used a machine learning algorithm to predict splice junctions from a training set of positive controls [22]. SpliceMap used flanking bases of splice site to locate potential splice sites [23], but the Mapsplice algorithm detects splice junctions without any dependence on splice site features [24]. The TopHat algorithm pairs candidate exons and evaluates the alignment of reads to such candidates [25]. GSNAP has the advantage of detecting both short- and long-distance splicing, including interchromosomal splicing [26]. PALMapper combines the read mapper GenomeMapper with the spliced aligner QPALMA [27]. In this study, we took a similar approach to TopHat. However, instead of pairing all potential exons, we only used exon-pairs that are compatible in phases. A phase indicates the position within a codon where an exon ends or starts [17]. Phase 0 indicates that an exon ends and starts after a codon ends or starts; phase 1 indicates that an exon ends and starts between the first and second base of a codon; and phase 2 indicates that an exon ends and starts between the second and third bases of a codon. Only combinations of exons that are in phase would not create any frame-shift after translation. In addition, in order to only identify those novel splicing events, we built a putative novel exon-exon junctional databases excluding all known exon-exon junctions annotated in all public domains including NCBI and EC genes [17]. Aligning of sequenced reads to this dataset would allow us to identify only novel splicing events that were not previously reported in EST sequencing or annotated in the public domains. Reads overlapping at least 8 base stretches from either side of junctions were considered putative splicing events. Furthermore, we restricted ourselves in identifying only those differentially expressed novel splicing events. The expression level of identified novel exon-exon junctions was evaluated by TPM (Tag per million) value. The aforementioned statistical methods were used to determine the expression difference of novel exon-exon junctions between HCC tissues and adjacent non-cancerous tissues.

### Validation of differentially expressed genes, exons and novel exon-exon junctions

Quantitative reverse transcription polymerase chain reaction (qRT-PCR) was used for the validation of differentially expressed genes, exons and novel exon-exon junctions. Seventy-six pairs of samples including 6 used for sequencing were applied for validation of DEG, while 20 pairs of samples were used for the validation of differentially expressed exons and novel exon-exon junctions. First, total RNA was extracted from tissue samples using E.Z.N.A. Mag-Bind Total RNA Kit I (Omega Bio-Tek, USA) following the protocol of manufacturer and then cDNA were synthesized from the total RNA using PrimeScript RT reagent kit (Takara, Japan). For the validation of DEGs, six genes were randomly picked from top 100 DEG list. They are *ANLN* (Anillin, actin binding protein), *GTSE1* (G-2 and S-phase expressed 1), *KIF14* (Kinesin family member 14), *MARCO* (Macrophage receptor with collagenous), *PTIGS* (prostaglandin I2 (prostacyclin) synthase), and *TFPI2* (Tissue factor pathway inhibitor 2). For the validation of differently expressed exons, 3 representative genes (*CFTR* of pattern 1, *CCDC50* of pattern 2, *SIGLEC11* of pattern 3) were chosen to illustrate 3 relationship patterns between DEEs and DEGs. The expression level of one pair of exons for each representative gene was compared between HCC tissues and adjacent non-cancerous tissues. The expression ratio for both exons of the same representative gene was also determined in HCC and adjacent non-cancerous tissues. For the validation of differently expressed novel exon-exon junction, the candidate list was first manually checked to investigate the read coverage and sequence overlaps at the novel exon-exon junctions. Six novel exon-exon junctions with best read coverage and sequence overlaps were selected, among which 4 were up-regulated and 2 were down-regulated in HCC tissues compared with adjacent tissues. For the validation of DEGs, Taqman assay approach (Premix Ex Taq™ kit) (Takara, Japan) was used. For the validation of DEEs, SYBR Green-based qRT-PCR approach was used with the SYBR Premix Ex Taq™ II kit (Takara, Japan). MX3005P QPCR System (Stratagene, USA) was used for quantitative RT-PCR according to the manufacturer's instructions. The primers and probes for Taqman approach were custom-designed and synthesized by Genecore Inc. (Shanghai, China). The PCR primers for SYBR Green-based approach were designed using Primer Premier 5.0 software (PREMIER Biosoft International, Palo Alto, CA, USA) and synthesized by Invitrogen Inc. (shanghai, China). The sequences of primers and probes, and the conditions of qRT-PCR reactions were listed in Table S2. The *HPRT1* gene was used as internal control. The relative expression level was determined using delta-delta CT method as previously described [28].

## Results

### RNA-seq analysis of 10 matched pairs of HCC and adjacent non-cancerous tissues

We performed RNA-seq on 10 pairs of matched HCC and adjacent non-cancerous tissues from Chinese HCC patients using the Illumina platform. The characteristics of the data were summarized in Table 1. On average, about 21.6 million sequencing reads and 10.6 million aligned reads were obtained for samples sequenced on one lane. It was commonly reported in the RNA-seq papers that a significant percentage of the sequence reads could not be mapped to the transcribed database [29]. There are several reasons for this, including 1) SNPs or other types of genetic variations; 2) repetitive elements and tandem repeats. 3) novel isoforms, splicing events or novel transcripts. Additionally, the transcript database that we used does not contain some RNA species such as U6 RNAs, small nucleolar RNAs, long non-coding RNA (lncRNAs) etc. Finally, there could be unspliced RNAs or genomic DNA contaminations. The average numbers of mapped genes, mapped transcripts or mapped exons were 24,356, 45,787 and 224,289, respectively. For each individual sample, we were able to identify about 64% of all the annotated genes that are expected to be almost equal to all detectable expressed genes in a given tissue. In the ENSEMBL database, the human database (Homo_sapiens.GRCh37.55.cdna.all.fa) includes 37,874 gene entries (ENSEMBL genes) and 95,605 transcript entries (ENSEMBL transcripts). In total from the 10 pair samples, we generated about 269 million (268,557,458) of aligned reads, which to our knowledge is the largest database representing transcripts expressed in HCC tissues. Combining all 10 paired samples, we were able to identify 33,262 genes (87.8% of all ENSEMBL genes) with one or more tags (Table S3). Furthermore, we calculated the average number of sequence tags that a gene has for the 20 samples, and we found that 17017 genes (51.2% of all ENSEMBL genes) have more than 10 mapped tags per kilobase (kb) of the transcript length and 24,062 genes (63.5%) with more than 3 RPKM. For the transcripts, we were able to identify 81.87% of (78,273 of 95,605, total entries in Homo-sapiens.GRCh37.55.cdna.all.fa) transcripts.

**Table 1:** Summary of sequencing data for 10 paired HCC samples.

Patients ID	Sequencing lanes(T/NT)	Total reads(T/NT)	Aligned reads(T/NT)	Number of mapped Genes(T/NT)	Number of mapped Transcripts(T/NT)	Number of mapped Exons(T/NT)
A05	1/1	20996588/22432079	10249035/12311804	25526/24104	46128/45190	237072/242629
A19	1/1	23586239/20834934	11485721/8843629	22127/22053	41210/39098	225402/205872
A31	1/1	23563690/21130309	11100731/10055694	25083/23924	47350/44033	250004/234018
A35	1/1	23060264/21127532	12602425/9179290	23447/22013	44553/38503	238811/195939
A57	1/1	21205625/20862092	10696149/13342966	23525/23676	43603/44300	237896/239578
A60	1/1	23315039/23481219	14396820/13525402	22706/23695	43207/44239	238530/241133
A63	1/1	20584096/20932296	11021167/8609693	21846/26041	41333/47528	226128/251622
A82	1/1	21068533/20378488	9967105/8401477	23354/25311	42401/45469	226371/220435
A13	2/2	39690126/40643375	13109377/18295444	29700/26164	57579/52158	267445/264099
A39	3/3	64805390/64921421	30429840/30933689	26891/25943	55220/52655	265052/256931

Notes: T/NT, tumor/non-tumor; Aligned reads were defined as those mapped to reference transcripts.

**Figure 1:**
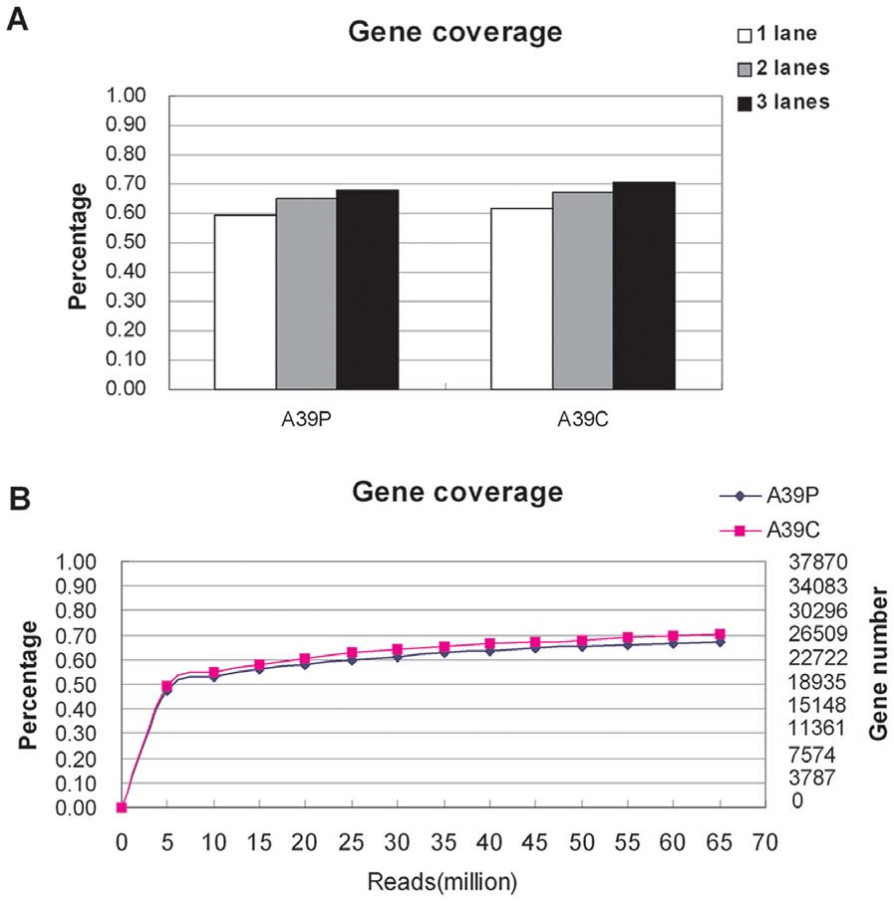
Assessment of sequencing depth required for transcriptome analysis. (A) Gene coverage of three sequential runs for sample A39P and A39C. (B) Gene coverage for 13 different bin size of million reads randomly chosen from the total sequenced reads of sample A39P or A39C.

To assess the depth of sequencing coverage required for transcriptome analysis, we re-sequenced one paired samples (A13C and A13P) on two lanes and another paired samples (A39C and A39P) on three lanes. Correlation analysis showed a good reproducibility between replicates with all Pearson Correlation coefficient (PCC) values>0.99 (Table S4). Our results indicated that the number of mapped genes or exons for paired samples (A39C and A39P) was not significantly increased for sequenced samples on 2 or 3 sequencing lanes, compared with those on one sequencing lane (Fig. 1A). In addition, because of the random sampling nature of the next-generation sequencing technology, we sought to use a random sampling approach to determine how many millions of sequence reads were sufficient to identify almost all genes expressed in a given tissue. The random sampling process was done using the raw reads, not mapped reads. We randomly sampled 5 to 65 million (using a step increment of 5 million) reads from the total raw reads that we have accumulated for sample A39P or A39C, and found that about 15 million raw reads were sufficient to identify more than 50% (21,170 genes, 56%, i.e. 21,170/37,874) of the annotated genes, which is almost equal to all detectable expressed genes in a given human tissue based on estimation from two large scale transcriptome studies [30], [31]. The gene coverage increased much slowly when the read size was larger than 15 million (Fig. 1B), suggesting that using a single sequencing lane (which can generate about 20 million raw reads) for each sample is sufficient for the transcriptome analysis when considering cost and benefit.

### RNA-seq data were comparable to the array data for Chinese HCC

**Figure 2:**
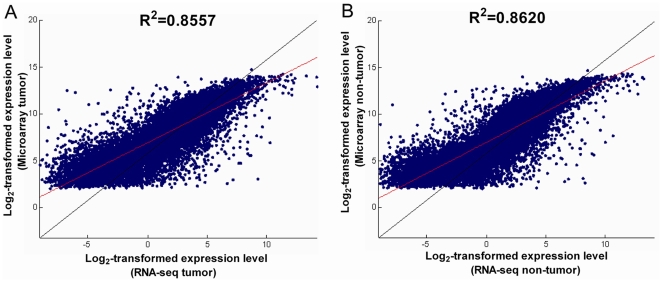
Correlation analysis of log_2_-transformed gene expression level between RNA-seq data and public microarray data (GSE22058) in (A) pooled tumor tissues and (B) non-tumor tissues. Red line stands for the best fit line, and the black line stands for the 45 degree reference line.

To assess the quality of RNA-seq data and see whether it has advantages over array-based approaches, our RNA-seq data was compared with the public microarray data (GSE22058). We performed correlation analysis of log_2_-transformed average gene expression level for common genes between the RNA-seq data and the microarray data. We found that the PCC was 0.8557 and 0.8620 for the pooled HCC tissues and pooled adjacent non-cancerous tissues, respectively, suggesting a high correlation between the two platforms. This observation was consistent with previous report that RNA-seq data is comparable to the array data [9]. In addition, we found that RNA-seq data captured a much wider dynamic range of expression level (tumor: −9.0737 to 14.2033; non-tumor: −10.4580 to15.6266) than array data (tumor: 2.0721 to 14.7653; non-tumor: 2.0358 to 14.6850) in log scales (Fig. 2A and 2B).

### Identification of differential expression at gene levels

**Figure 3:**
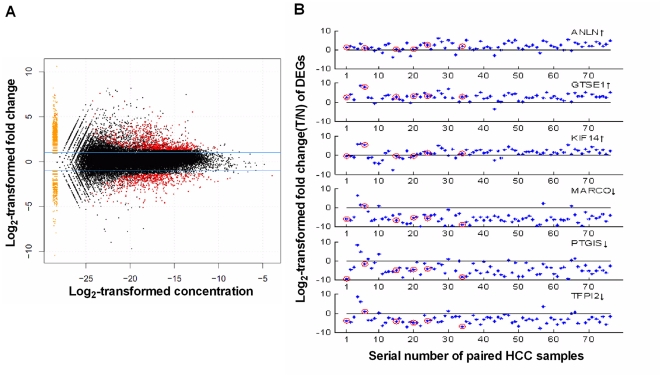
The distribution of differentially expressed genes in MA plot (A) and their validation by qRT-PCR (B). Fold change (T/NT) of the expression level for a given gene was defined as the read counts of the gene in the tumor sample (T)/the read counts in the non-tumor sample (NT); Concentration for a given gene was defined as the read counts of gene/the total read counts in HCC sample+the read counts of the gene/the total read counts in non-cancerous samples. Each dot represents a gene. Yellow dots represent the genes with no counts in either HCC tumor or non-cancerous tissues. Red dots represent the genes with significantly differential expression. Red rings represent those samples also used for RNA-seq analysis.

**Figure 4:**
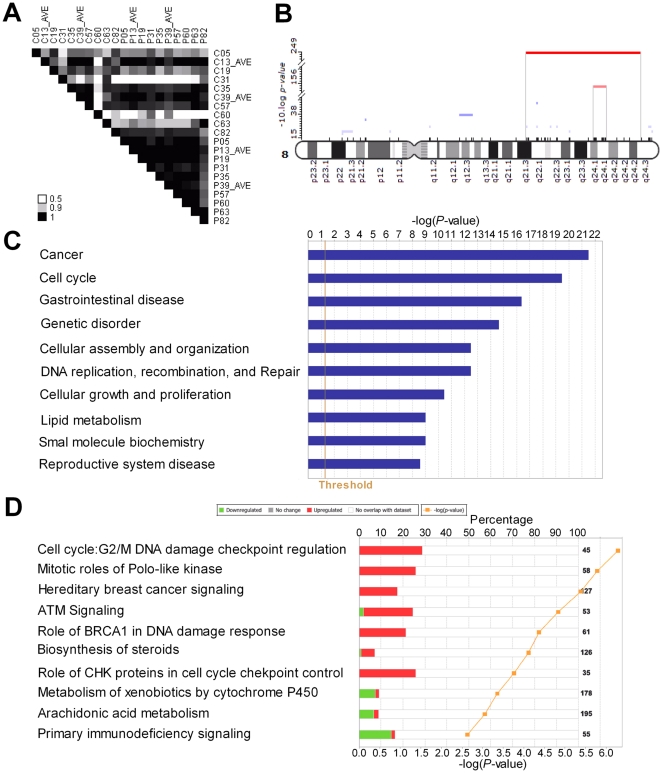
Functional annotation of DEG. (A) Analysis of homogeneity for HCC and adjacent non-cancerous tissues using PCC values. The PCC values of gene expression between different samples were represented by different colors: bigger value corresponding to darker color. AVE: Average raw reads number for each gene from re-sequenced sample. The character C represents cancerous tissue sample and P for peri-cancerous tissue sample. (B) Enrichment regions of up-regulated genes on Chromosome 8. (C)Top 10 bio-function terms enriched by DEGs and (D) top 10 canonical pathways enriched by DEGs. Percentage represents the ratio of the number of mapped genes to all genes in a pathway from IPA. Threshold was set (*P*<0.05 or −log (p)>1.3) by Fisher's Exact Test.

We identified a total of 1,378 significantly differently expressed genes (DEGs) with 808 up-regulations and 570 down-regulations in HCC, when compared with non-cancerous adjacent tissues (Table S5). The numbers of DEGs seem to be evenly distributed at different expression abundance levels (Fig. 3A). The top 30 DEGs, which were identified based on descending ranking of FDR from the edgeR program, were listed in Table S6. A more systematic comparison of these 30 DEGs in 3 public HCC databases revealed that 22 DEGs (73.3%) were identified to be DEGs in at least one of the three databases. Validation analysis using quantitative RT-PCR for randomly picked six DEGs demonstrated the consistent over-expression for *ANLN*, *GTSE1* and *KIF14* and the consistent under-expression for *MARCO*, *PTGIS* and *TFPI2* in HCC (Fig. 3B). Interestingly, we found that the average PCC (0.9083) for any two adjacent non-cancerous tissues was significantly higher than that (0.7218) for any two cancer tissues or that (0.7336) for any cancer and non-cancerous tissue pairs, suggesting a higher degree of biological heterogeneity in HCC tissues compared with non-cancerous adjacent tissues (Fig. 4A.)

The chromosome regions enriched by DEGs were analyzed using PGE software. Using a much stringent FDR value of <0.001), up-regulated genes or down-regulated genes in tumor tissues were significantly enriched at 18 or 9 chromosome regions respectively. In 14 non-redundant enriched regions (4 of the 18 are redundant) that were enriched by the up-regulated genes in HCC (Table S7), eleven of them (about 73%) were reported to be amplified in HCCs and four of them were not reported in the literature. A similar literature searches identified 6 out of 9 regions enriched by down-regulated genes in HCC tissues showed allele loss or rearrangement that could explain the down-regulation of genes. Three of the 9 regions did not have literature report of allele losses (Table S8). The most significant enrichment was located at chromosome 8q21.3–24.3 (90839173–141714827) with up-regulation of 44 genes among a total of 156 in this region, which could be further refined to chromosome 8q24.1 (120812196–126448543) with up-regulation of 18 genes among 30 (Fig. 4B). Additionally, we also found that 1q22–23 was significantly enriched by over-expressed genes in HCC, with 9 of 37 genes in this region up-regulated. A smaller region within 1q22–23 included 5 up-regulated genes out of 10 in total. These regions were also identified as chromosome aberrations in previously study [32], [33]. Furthermore, we found many novel potential chromosome aberrations including 15q15.1 that was enriched with up-regulated genes, and 10q23 and 9q34 that were enriched with down-regulated genes in tumors. These regions were not found in previous array-CGH analysis of HCC [2].

Using the IPA software package, we identified 54 significant bio-function terms and 41 significant canonical pathways (Fisher's Exact Test, *P*<0.05) (Table S9 and Table S10). The top 10 significant bio-function terms and canonical pathways were shown in Fig. 4C and 4D, with cancer and G2/M DNA damage checkpoint regulation in cell cycle ranking the highest. Most of other bio-function terms and pathways were also closely related to tumorigenesis, such as cell cycle, cell growth and proliferation, lipid metabolism, DNA replication and repair.

Finally, we analyzed the DEGs using a tool GSEA and found that the top enriched set is the targets of miR199A and B (*P* = 0.002) when using C3.all.v2.5 gene sets (C3 represents all motif gene sets including microRNA targets and transcriptional factor targets). The genes of *BCAM* (basal cell adhesion molecule), *NCOA2* (nuclear receptor coactivator 2) and *NPAS2* (neuronal PAS domain protein 2) were listed in this gene set that were up-regulated in HCC tissues when compared with the adjacent non-cancerous tissues. When C2.all.v.2.5 gene sets (C2 represents all curated genes) were used, the top gene sets enriched by up-regulated DEGs in cancer is the SMITH_LIVER_CANCER gene set (*P* = 0.0039), which contains potential marker genes specifically up-regulated in the majority of HCV-related HCC [34]. Other enriched gene sets with *P* value<0.05 were listed in Table S11.

### Complex pattern of gene regulation revealed by RNA-seq coupled with exon-level analysis

**Figure 5:**
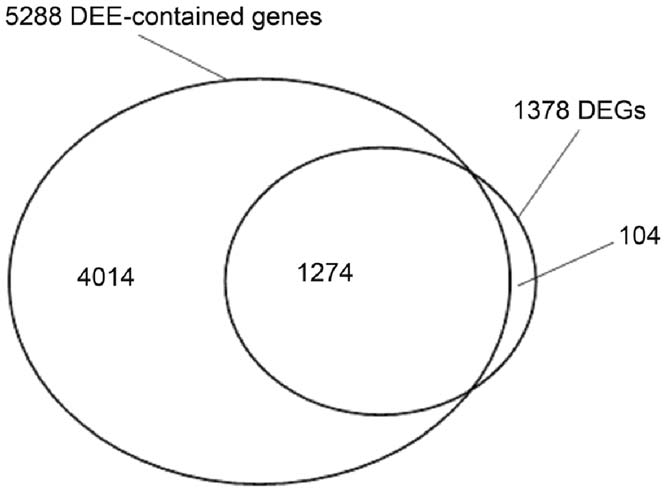
Venn diagram of intersection between 1378 DEGs and 5288 DEE-contained genes.

We analyzed differential expression at exon levels by mapping sequenced reads to each individual exon of genes. Totally, about 85.66% of (329891/385122) all annotated exons in the human genome were mapped when combining the 10 pairs of samples. Using the same statistical analysis procedures aforementioned for DEGs, we obtained a total of 24, 338 differentially expressed exons (DEEs) between HCC and adjacent non-cancerous tissues (data available when required), which correspond to 5288 genes. Among these, 1274 of these genes were also identified by the gene level analysis (1378 DEGs genes identified at gene levels). However, 4014 of these genes showing differential expression at the exon levels were not found in the DEGs that were identified at the gene levels, suggesting that RNA-seq data combined with exon level analysis might capture a much more complex landscape of differential gene expressions. A Venn diagram illustrating the overlaps was shown in Fig. 5. Since the length of the tag sequence we used for the alignment is 36 base pair, and we allowed up to two mismatches (2/36, i.e. 5.56%), unless the HCC cancer transcriptome has a mutation rate greater than 5.56%, there would not be any bias of exon-level expression caused by the sequence differences in the tumor and normal tissues. We have checked the mutation rate of HCC and found that it is similar to other types of cancers. We found that there are 743 to 3050 SNPs per transcriptome among the 20 samples and there is no significant difference in the number of SNPs between the HCC and the adjacent tissue group (Data not shown). Assuming that we covered about 2% of the genome (about 2% of the genome are exons), then the polymorphism (including somatic mutation) rate would be around 0.0012–0.0051%.

**Figure 6:**
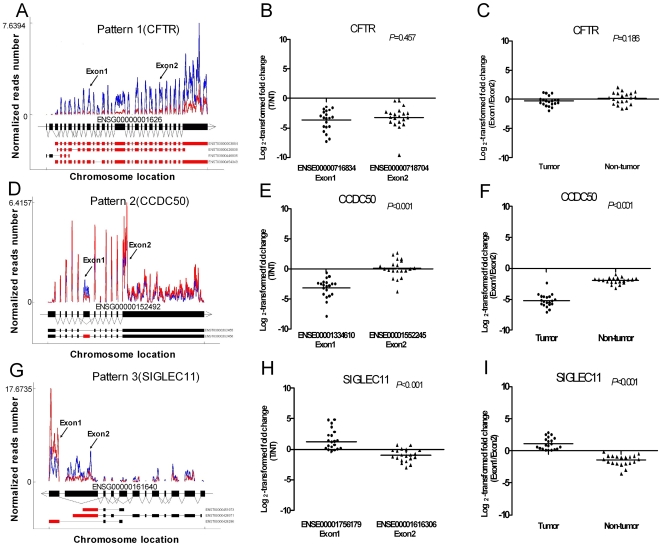
Analysis and validation of DEEs. (A), (D) and (G): Schematic diagrams of 3 representative genes (*CFTR*, *CCDC50*, *SIGLEC11*) illustrating 3 relationship patterns between DEEs and DEGs. The structure of genes, together with all annotated exons and transcripts, were shown at the bottom. The DEEs were highlighted in red on annotated exons of transcripts showing at the bottom. Red curve represents the normalized read counts for HCC tissues and blue curve represents the normalized read counts for adjacent non-cancerous tissues. (B), (E) and (H): Comparison of expression levels of DEEs in three representative genes between HCC and adjacent non-cancerous tissues. (C), (F) and (I): Comparison of expression ratio for different DEEs of the same representative gene in HCC and adjacent non-cancerous tissues. T: tumor NT: Non-tumor.

To gain detailed understanding of the differential expression at exon levels and the relationship of differential expression analysis between gene level and exon level, we plotted the read counts for each exon of 5288 genes. Several patterns emerged. Representative expression patterns were illustrated in Fig. 6. The most common pattern (pattern 1) (Fig. 6A) showed consistent differential patterns for all exons of DEGs. These genes would be identified as differentially expressed by either exon-level or gene-level analytical approaches. A more interesting pattern is illustrated by pattern 2 (Fig. 6D) where selected exons were differentially expressed while the rest of the exons were not differentially expressed. However, the direction of expression changes (up or down regulation) is the same for the differentially expressed exons (DEEs). Finally, there are more complex patterns (pattern 3) (Fig. 6G) where both up- and down-regulated DEEs exist for the same gene, suggesting a complex regulation mechanism. We have retrieved mapped reads from the exon-exon junction alignments for 3 exampled genes. We did observe the sequence reads that mapped to the exon-exon junctions and found different junction alignments for 3 exampled genes in HCC and non-cancerous tissues. These results were in consistence with corresponding DEEs and evidently support the alternative splicing as the explanation for the exon expression difference (data not shown).

We further confirmed the existence of all these patterns by quantitative RT-PCR using 20 HCC patients (Fig. 6B, C, E, F, H, I). *CFTR* (cystic fibrosis transmembrane conductance regulator) represents pattern 1 of DEGs. We found that the two DEEs (ENSE00000718634, ENSE00000718704) of this gene were both significantly down-regulated to similar extent in tumor tissues when compared to non-tumor tissues (both *P*<0.001) (Fig. 6B). However, the ratio of these two DEEs showed no difference (*P* = 0.185) between tumor and non-tumor tissues (Fig. 6C). The gene *CCDC50* (coiled-coil domain containing 50) represents pattern 2. One DEE (ENSE00001334610) of *CCDC50* in pattern 2 was significantly down-regulated in tumor tissue (*P*<0.001), while another DEE (ENSE00001552245) exhibited no significant expression difference between tumor and non-tumor tissues (*P* = 0.9496) (Fig. 6E). The ratios of expression level of two exons in *CCDC50* were clearly higher in tumor tissues than that in non-tumor tissues [median (range): −5.2775(−7.045, −2.335) *vs.* −1.8725(−3.175, −0.97) *P*<0.001] (Fig. 6F). Finally, the gene *SIGLEC11* (sialic acid binding Ig-like lectin 11) represents pattern 3. One DEE (ENSE00001756179) of *SIGLEC11* was significantly up-regulated in tumor tissue (*P*<0.001), while another DEE (ENSE00001616306) was significantly down-regulated (*P*<0.001) (Fig. 6H). The ratios of expression level of two DEEs in *SIGLEC11* were considerably different between tumor and non-tumor tissues [median (range): 0.8475(0.1, 2.875) *vs.* −1.3025(−3.610, −0.185) *P*<0.001] (Fig. 6I).

### Identification of novel differently expressed splicing variants

**Figure 7:**
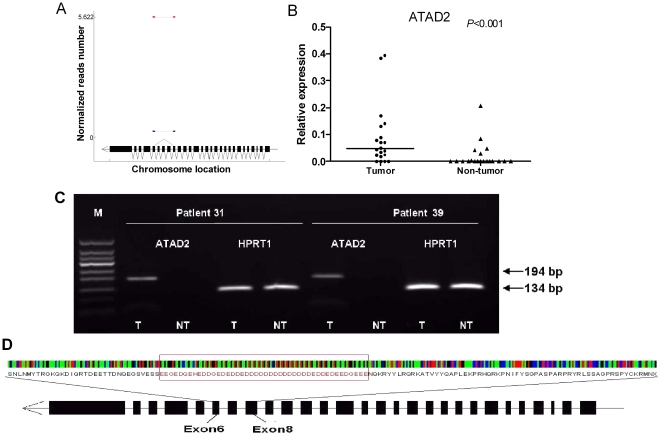
Analysis and confirmation of a differently expressed novel exon-exon junction identified for the *ATAD2* gene. (A) A schematic diagram of the differently expressed novel junction in *ATAD2*. Red line represents tumor tissues and blue line represents adjacent non-cancerous tissues. The structure of gene was plotted at the bottom. The connecting lines below exons represent all known annotated junctions. The red connecting line above exons represents the predicted novel exon-exon junction. (B) Relative expression of the novel junction of *ATAD2* in HCC and non-cancerous tissues. (C) RT-PCR analysis on agrose gel electrophoresis for the novel junction in HCC and non-cancerous tissues from 2 HCC patients. HPRT1 was used as the internal control. (D) A schematic diagram of amino acid sequence for exon 6–8 in *ATAD2* gene.

We explored the possibility of RNA-seq in identifying novel spliced transcripts consisting of novel splicing events of known annotated exons using a similar approach to TopHat. Finally, we identified 61 differently expressed novel exon-exon junctions between HCC and adjacent non-cancerous tissues in 29 genes (Table S12). We selected six novel junctions for validation in 20 HCC patients using qRT-PCR and agrose gel electrophoresis. However, we were only able to confirm one novel junction in *ATAD2* (ATPase family, AAA domain containing 2) gene in the samples. Furthermore, the novel splicing variant of *ATAD2* was detected to be with significantly higher frequency and abundance in HCC compared to adjacent non-cancerous tissues (in only 5 adjacent non-cancerous tissues but in all 20 HCC tissues, p<0.001) (Fig. 7B). PCR confirmation analysis showed that the novel splicing event was only found in tumor samples but not in adjacent tissues (Fig. 7C).

## Discussion

In this study, we conducted the comprehensive transcriptome sequencing for 10 HCC and 10 non-cancerous adjacent liver tissues (matched pairs). An analysis using repeated sequencing runs and random sampling simulations showed that about 15 million raw reads were sufficient to cover 50% (21,170) of all the annotated genes. Jongeneel *et al.* [31] comprehensively analyzed the expression of mRNAs using massively parallel signature sequencing (MPSS) in human tissues and concluded that about 50% of genes in the human genome are expressed in a given tissue. Sugarbaker *et al.* [35] showed that about 15000 genes could be detected by one or more reads using about 2.5–3 millions reads and they demonstrated that the slope of gene coverage reaches a plateau after 5 million sequence reads. Since not all genes are expressed at given tissues, our ability to sequence 17K genes at sequence depth of >10 tags per gene was quite good. We believe that this sequence depth allowed meaningful comparison of at least 51.2% of the genes. Therefore, our sequencing reads of >20 million for each sample should be sufficient for transcriptome analysis considering cost and benefit. Furthermore, a comparison between our RNA-seq data and the public microarray data illustrated that deep sequencing could detect a much wider dynamic range of gene expression than microarrays. Expression profiling analysis by arrays is limited by its low sensitivity due to background hybridization and sometimes reduced specificity due to cross-hybridization of probes and targets [8], [9]. RNA-seq is a more sensitive technology and it is thus not surprising to see that we identified additional novel DEGs. To the best of our knowledge, this represents the most comprehensive characterization of the transcriptome of liver tissues, which is critical to understand the disease at system-wide levels as any missing data will create biased view of the system.

We first analyzed the expression difference at gene levels between HCC and matched non-cancerous adjacent tissues and identified 1378 differentially expressed genes (DEGs), many of which were identified as differentially expressed in previous studies such as *GPC3* (glypican 3) [36], *TERT* (telomerase reverse transcriptase) [37], *SPINK1* (serine peptidase inhibitor, Kazal type 1) [38] and *ESM1*(endothelial cell-specific molecule 1) [39]. Many additional novel and un-annotated genes were identified, suggesting the power and sensitivity of the RNA-seq based approach for expression profiling. Functional analyses indicated that 1378 DEGs were mostly enriched in the 54 bio-function terms and 41 canonical pathways, which provided important clues for understanding the molecular mechanisms of HCC pathogenesis. Among these pathways, most of them have been previously characterized as onco-signal pathways in HCC pathogenesis like cell cycle pathway, molecular signaling pathways of *MAPK*, *p53*, *BRCA1*, although several pathways were only indicated previously on the basis of very few mapped genes [3], [40]. Actually, comparing to previous reports, we have identified more mapped genes with significant expression changes in several pathways (data not shown). For example, more members of cyclin family and MAPK (Mitogen-Activated Protein Kinase)-related family were found to be up-regulated in HCC tissues compared with adjacent non-cancerous tissues.

The DEGs were also significantly enriched for a number of fundamental and conserved metabolism processes, such as biosynthesis of steroids, retinol and fatty acid. Particular interestingly is the observation that many members of cytochrome P450 (CYP450) family were significantly down-regulated in HCC tissues, which is consistent with reported data on reduced activities and down-regulated expression of various P450 molecules in the liver [41]. This is not surprising because liver cancer could cause the loss of normal hepatic cells that produce P450 enzymes. Another noteworthy finding was the down-regulation of many immune-related genes in HCC tissues, such as heavy and light chain genes of immunoglobulin and immunoglobulin receptor genes. This could be caused by the fact that the tumor tissues might have fewer lymphocytes than the adjacent non-cancerous liver tissues as HCC often have lymphocyte infiltration [42]. Finally, we also found that the targets of miR199A and B were enriched in the DEGs, including *BCAM*, *NCOA2* and *NPAS2*, all of which were up-regulated in HCC compared to adjacent tissues. The role of the three up-regulated genes in HCC remains to be studied. Recently, Yeligar *et al.* [43] showed that miR199 was inducible by ethanol and that its down-regulation may contribute to augmented HIF-1alpha and ET-1 expression. Taking together, RNA-seq analysis revealed that malignant transformation of hepatocytes might involve the perturbation of multiple important cellular pathways including cell growth-related pathways, metabolism-related processes, immune-related and micro-RNA regulated pathways.

Accumulated somatic genetic alteration is a hallmark of cancer genome and a variety of chromosome aberrations have been identified in HCC tissues through traditional CGH and array-CGH methods with limited resolutions [2]. Microarray-based expression profiling data were also intended to use for the screening of genetic aberrations at the level of chromosome [44]. Several published array CGH data and comparative genomic microarray analysis (CGMA) have identified the amplification of chromosomal 8q, 1q, 20q, 17q and the deletion of chromosome 4q, 8p, 13q, 16q, 17p as frequently events in HCC [32], [33], [45]. In the present study, we for first time attempted to make full use of RNA-seq data for the screening of chromosomal aberrations in HCC. Our data indicated that many of chromosomal aberrations predicted in our study were almost identical to what was previously reported [2], such as the amplification of 8q24. The chromosome region of 8q24 harbors a number of potential onco-genes, including *c-Myc* [46], *CCNE2* [47], and *RIPK2* [48]. Another chromosomal segment 1q22 was found to be enriched by many genes that were up-regulated in HCC compared with adjacent tissues. Wong *et al.* [49] identified that this segment was amplified in HCC tissues. In contrast, chromosomal regions 4q12, 4q23 and 4q32 were enriched by genes that were down-regulated in HCC when compared with adjacent tissues. These regions were loci with loss of heterozygosity (LOH) in HCC [44]. These results suggested that the deep sequencing might be feasible and reliable for screening of chromosome aberrations with higher resolution and sensitivity. Except for the findings as previously reported, we also predicted several novel potential chromosome aberrations, which were not found in previous array-based studies. Among them, a representative chromosome regions were located on the chromosome 15.1, which were enriched by a number of up-regulated carcinogenesis-related genes, such as *OIP5*, a potential cancer therapeutic target [50], and *PAK6*, a member of the p21-activated kinase (PAK) family of serine/threonine kinases, may play roles in the regulation of cell motility and in stress responses [51]. Our findings gave a deep insight into the HCC pathogenesis at the level of chromosome. Further studies are still needed for the detailed explanation of chromosome aberrations in hepatocarcinogenesis.

Alternative splicing is a process in which cells can selectively include different regions of pre-mRNA during RNA processing and it was found to be implicated in carcinogenesis [52]. However, the complex nature of alternative splicing makes its discovery not easy. Previous efforts using exon arrays have achieved only limited success [53]. Taking advantage of the RNA-seq technology, we were able to analyze differential expression at the exon levels, providing a way to understand fine scale regulation of gene expression at alternative transcript level for the same gene. We first characterized in detail three patterns of regulation and confirmed the existence of all these three patterns (Fig. 6). Made possible due to the power of the RNA-seq technology, our database provides the most comprehensive characterization of relationship between gene regulation at gene and exon levels. It will become an important resource for the scientific community to understand the complex molecular mechanism of hepatocarcinogenesis.

Our data set also allows us to identify novel splicing events (Fig. 7) that are not previously annotated or reported even by the huge EST sequencing efforts. For example, we identified a novel splicing variant for *ATAD2* (ATPase family, AAA domain containing 2) that was over expressed in HCC samples. ATAD2 is a very important molecule as it is a cofactor for MYC, AR (androgen receptor) and ERalpha and the gene itself is regulated by both estrogens and androgens [54], [55]. ATAD2 maps to chromosome 8q24, a region that is frequently found to be amplified in cancers including in HCC as we described above. It has 28 exons and the splicing events would create a transcript lacking exon 6–8 and result in a protein isoform that misses 136 amino acids from the original ATAD2 protein. A functional domain search using the InterPro scan program (http://www.ebi.ac.uk/Tools/pfa/iprscan/) revealed that the skipping region matches to the pfam domain PF05764 (YL1 nuclear protein), which has DNA-binding properties. However, the ATP binding domain (at amino acid position 467–474) of the ATAD2 protein was not affected by this exon skipping event. In addition, we found that this skipping region contains a trinucleotide repeat that translates into a peptide sequence characterized by 24 aspartic acids (D) and 15 glutamic acids (E), both of which are negatively charged amino acids (Fig. 7D). To date, the biological significance of skipping this repeat sequence and the functional of the novel ATAD2 isoform remains to be determined, and further investigation is warranted.

A limitation of our dataset in analyzing splicing events is that we only used 36-nucleotide sequence reads for the alignment. In the analysis pipeline, only tags with more than 8 nucleotides (nt) spanning each of the two joining exons were considered as evidence for a putative splicing event. Although this approach could be highly sensitive, it also results in high false positive rate. Future work using longer sequencing reads or using paired-end sequencing will help to alleviate the problem.

In conclusion, this study for the first time utilized next generation sequencing platform to comprehensively characterize the HBV-related HCC transcriptome. The full characteriztion of the landscapes of the HCC transctiptome provides the basis for an understanding of the molecular mechanisms of HCC pathogenesis at system-wide levels. Future research works based on our findings may speed up the discovery of novel biomarkers and drug targets for improving diagnosis and therapy of HCC.

## Supporting Information

Table S1 **Characteristics of HCC patients used for RNA-seq analysis.**
(XLS)

Table S2 **Sequence of primers or probes and PCR conditions for qRT-PCR validation.**
(XLS)

Table S3 **The RPKM value of each gene for 10 paired samples.**
(XLS)

Table S4 **PCC values of raw reads-based gene expression levels between replicates of sequenced sample.**
(XLS)

Table S5 **Differently expressed genes between HCC and adjacent non-cancerous tissues.**
(XLS)

Table S6 **Top 30 of differently expressed genes.**
(XLS)

Table S7 **Chromosomal regions enriched by up-regulated genes in HCC tissues.**
(XLS)

Table S8 **Chromosomal regions enriched by down-regulated genes in HCC tissues.**
(XLS)

Table S9 **Significant bio-function terms enriched by differently expressed genes.**
(XLS)

Table S10 **Significant canonical pathways enriched by differently expressed genes.**
(XLS)

Table S11 **Gene sets enriched in DEGs from RNA-seq data.**
(XLS)

Table S12 **Significant differently expressed novel exon-exon junction.**
(XLS)
